# Paraquat Induces Lung Injury *via* miR-199-Mediated SET in a Mouse Model

**DOI:** 10.3389/fphar.2022.856441

**Published:** 2022-04-01

**Authors:** Quan Cai, Yan Jin, Ziyi Jia, Zhi Liu

**Affiliations:** Department of Emergency, The First Hospital of China Medical University, Shenyang, China

**Keywords:** paraquat, lung injury, poisoning, SET gene, miR-199

## Abstract

**Objective:** To explore the molecular mechanism of lung injury caused by paraquat (PQ) poisoning by investigating miR-199-mediated SET.

**Methods:** A paraquat poisoning model was established in C57BL/6 male mice via intraperitoneal injection of paraquat. The mice were transfected with miR-199 siRNA and or mimic. After 14 days of treatment, pathophysiological changes of the lung were observed and lung tissue was analyzed via Hematoxylin-Eosin staining. The levels of miR-199, SETs, surfactant protein SP-A and SP-B, and inflammatory and oxidative factors were analyzed by qPCR, Western Blot, and ELISA kits.

**Results:** A acute lung-injury (ALI) model was established using PQ treatment and confirmed with edema of pulmonary endothelium with low electronic density of endothelial cytoplasm, presence of protein-rich fluid, and numerous erythrocytes in alveolar space, concentric figures of damaged tubular myelin, alveolar destruction, and increase in inflammatory cell numbers. Compared with the control group, miR-199 and SET levels were reduced in the PQ-treated group. miR-199 siRNA increased the SET level, inflammatory and oxidative levels, and reduced the levels of SP-A and SP-B, and miR-199 mimic reduced the SET level, inflammatory and oxidative levels, and increased the levels of SP-A and SP-B. PQ treatment reduced miR-199 level.

**Conclusion:** Paraquat induces ALI by affecting miR-199-mediated SET.

## Introduction

Paraquat (PQ) is a dichloride compound, a bipyridine quaternary ammonium herbicide with quick-acting and nonselective contact (Tsen et al., 2019). Many countries have banned the sale of PQ water solvents due to its high mortality rate accounting for 13% of all fatal cases caused by PQ poisoning. However, there are still PQ poisoning incidents (Sittipunt, 2005; Weng et al., 2017; Lin et al., 2021). The lung has the ability to actively take in PQ, so it becomes the most important target organ because of PQ poison (Jiang et al., 2019; SreeHarsha, 2020). The degree of lung damage directly determines the patient’s prognosis. Current studies suggest that the main molecular mechanism of PQ poisoning is the damage caused by excessive redox reactions and inflammatory responses (Pourgholamhossein et al., 2018; Mirzaee et al., 2019). For the lung, pulmonary edema and pulmonary hemorrhage may occur under the action of a large number of reactive oxygen species (ROS) in the early stage (Liu et al., 2018a). Acute lung injury (ALI) symptoms such as atelectasis and atelectasis can later progress to pulmonary interstitial fibrosis (Wang et al., 2019; Rashid et al., 2020). Respiratory failure caused by the two is the main cause of death (Bi et al., 2020; Johnson et al., 2020). Although some progress has been made in clinical and basic research based on oxidative stress and the pathogenesis of the inflammatory cascade in recent years, the clinical treatment effect is limited, and it has not really improved the survival rate of patients with PQ poisoning (Yeh et al., 2020). Considering the mechanism of PQ poison-caused ALI, the in-depth study of the molecular mechanism of ALI caused by PQ poisoning and exploring its potential mechanisms and targets have become very important.

miRNAs are small noncoding RNAs extending over 18–22 nucleotides and regarded as the modifiers of many respiratory diseases (Jiang et al., 2020). MiR-199 is a well-identified miRNA playing various roles in many physiological and pathological activities. The reduction in the level miR-199 has been found to protect sepsis-induced ALI by targeting SIRT1(Liu et al., 2018b), while the level of miR-199 is reduced in lipopolysaccharide-induced acute lung injury model (Park et al., 2018), suggesting miR-199 may affect ALI.

SET is a multi-functional protein that modulates various cell signaling pathways including nucleosome assembly and histone binding, and it has existed in many types of human tissues, especially the lung (Liang et al., 2021). SET protein has multiple functions, and oxidative stress and inflammatory activities may also lead to abnormal SET gene expression (Feng et al., 2017). Reduced expression of miR-199 can increase the levels of SET, suggesting SET may be a target of miR-199 (Chao et al., 2010). PQ treatment was found to stimulate miRNA profiling responses and changed their expression levels (Huang et al., 2014; Wang et al., 2018a). Therefore, PQ may cause ALI by affecting miR-199-mediated SET. On the other hand, surfactant protein (SP) is an important component of lung surfactant, including SP-A and SP-B (Yang et al., 2020). They play an important role in maintaining the physiological functions of the alveoli and mediating the immune balance of the local microenvironment. Numerous studies have also suggested that the content of SP has a significant decreasing trend in the case of ALI. All the related important molecules were explored in this study.

## Materials and Methods

### Experimental Animals

Thirty-two C57BL/6 male mice (6–8 weeks old, 18–22 g) were purchased from animal center of our organization. The mice were freely feed food and water that meet the standards, and lived in a clean environment with 12 h day/12 h night cycle in the room temperature at (22 ± 0.5)°C, humidity 55 ± 5%. All the processes of the experimental research are following the operating specifications of laboratory animals and approved by the animal research committee of our hospital.

### Establishment of Paraquat-Induced Acute Lung-Injury Mouse Model

The mice were injected intraperitoneally with PQ 40 mg/kg (PQ dissolved in sterile saline, 1 mg/ml). The mice in the control group were treated in the same way as the mice in the experimental group with equal volume of sterile saline. After 14 days exposure to PQ, four mice from each group were anaesthetized with pentobarbital (60 mg/kg) at each time, and the abdominal cavity was fully exposed and cut along both sides of the sternum stem. The heart and lung tissues were fully exposed, the abdominal aorta was dissociated, and warm saline with constant pressure from the right ventricle was infused through the pulmonary circulation, until the lungs turn white. The lung tissue was separated, and the left lung tissue was taken for subsequent analysis. The upper right lung was dehydrated in 4% paraformaldehyde and embedded in paraffin for pathological staining after sectioning. The lower right lung was stored at −80°C.

### Injection of miR-199 Mimic or siRNA Lipoplexes Into Mice

miR-199a mimic, 5′-CCC AGU​GUU​CAG​ACU​ACC​UGU​UC-3′; miR-199a siRNA,0 5′-CCC​AGU​GUU​CAG​ACU​ACC​UGU​UC-3′; and miR-199a blank control, 5′-CAG​UAC​UUU​UGU​GUA​GUA​CAA-3′; miR-199a mimic control, UUC​UCC​GAA​CGU​GUC​ACG​UTT; and miR-199a siRNA control, CAG​UAC​UUU​UGU​GUA​GUA​CAA were synthesized from Sangon Biotech (Shanghai, China). LPs were performed as follows: dioleoyl-tri-methylammonium propane (DOTAP) and cholesterol were dissolved in the dichloromethane solution according to the molar ratio of 1:1 (mol/mol). After being dissolved and mixed well, the mixture was drawn into a 20 ml round-bottomed flask. It took about 2 min to remove dichloromethane by rotary evaporation, and a thin film was formed on the wall of the bottle. Then, it was dissolved in 1 ml of nuclease-free water to form a mixture of DOTAP and cholesterol with a final concentration of 10 mM. The mixed solution was uniformly dispersed through an ultrasonic cleaner, filtered with a liposome extruder (100 nm filter membrane), and stored at 4°C. 1 µg of siRNA or mimic was dissolved in 66 μL of nuclease-free water, and protamine was added (calculated by mass ratio between siRNA and protamine), mixed gently, and incubated at room temperature for 10 min. The prepared liposome mixture should be mixed with an ultrasonic cleaner before use. A total of 60 μL of 10 mM liposome mixture was added to every 36 μg of protamine and left at room temperature for 5 min. Lipoplexes carrying 20 µg of miR-199 mimic or siRNA were injected into tail veins of mice.

### Animal Grouping

According to different treatments, C57BL/6 male mice were divided into eight groups (*n* = 4 for each group): blank control group (BG), PQ-treated group (PG, a paraquat poisoning model was established via intraperitoneal injection of paraquat), miR-199 siRNA control group (ICG), miR-199 mimic control group (MCG), miR-199 siRNA group (IG), PQ-treated miR-199 siRNA group (PIG), miR-199 mimic group (MG), and PQ-treated miR-199 mimic group (PMG).

### Hematoxylin-Eosin Staining

All mice were sacrificed by cervical dislocation after 24 h miR-199 mimic or siRNA transfection (Chernikov et al., 2017), and lung tissues were isolated from the mice. HE staining is one of the main stains in histopathology and the widely used method in medical judgement. HE staining was performed according to the previous report (Tianzhu et al., 2014) and pathological changes of lung tissues were observed. According to a previous report (Pua et al., 2005), mean linear intercept (MLI) was used to determine the ALI by overlaying a prearranged grid on the image, with set placed lines totaling 0.5 mm in length. Inflammatory cells were counted according to the histological observations with eosinophils, neutrophils, and mast cells (Corrêa et al., 2017). The number of inflammatory cells in ALI tissues on day 14 was counted in five random different fields under microscope, and average values in each field were calculated. Furthermore, the investigators were blinded to the treatment groups during examining slides/histopathology.

### qPCR

A total of 700 μL of QIAzol lysis solution was added to the grounded lung tissues in a 2 ml tube, and incubated for 5 min at room temperature (15–25°C). Total RNA was isolated according to kit instructions (B511321, Sangon Biotech, Shanghai, China). The concentration of RNA sample was measured by using nanodrop 2000. cDNA was synthesized using First Strand cDNA Synthesis Kit (Sangon Biotech, Shanghai, China). The primers (Table 1) were synthesized by Sangon Biotech (Shanghai, China). At the same time, the internal reference gene small nuclear RNA, U6, was synthesized. Following the steps in the miScript SYBR® Green PCR Kit, qPCR components were prepared as follows: 2 × QuantiTect SYBR Green PCR Master Mix, 10 × miScript, 10 × miScript Specific Primer, template cDNA, and RNase-free water. The thermal cycling conditions included 10 min at 95°C, and proceeded with 40 cycles of 95°C for 0.5 min and 60°C for 2 min.

**TABLE 1:** Primers were used in this study.

Gene	Forward primer	Reverse primer
miR-199	CCA​GTG​TTC​AGA​CTA​CCT​G	GAA​CAT​GTC​TGC​GTA​TCT​C
SET	TGA​CCC​GTC​TTC​AAA​GTC​CAC​C	AGC​ACC​TGC​GTC​AGA​ATG​GTC​A
U6	CTCGCTTCGGCAGCACAT	TTT​GCG​TGT​CAT​CCT​TGC​G

### Western Blot

The lung tissues were washed with PBS repeatedly for 2 to 3 times, and total protein was extracted with RIPA. The protein concentration was measured by the UV spectrophotometer Q5000. A 5 × SDS loading buffer was added to the supernatant after cell lysis and boiled at 100°C for 10 min. 40 μg of total protein sample was loaded onto the pre-configured 12% SDS gel, and SDS electrophoresis solution was added. Electrophoresis was performed at 80 V constant pressure electrophoresis for 30 min, then switched to 120 V constant pressure electrophoresis for 1 h. The PVDF membrane was activated with anhydrous methanol and put in the transfer solution, and let it stand for 3 min. Sandwich method was used to transfer the membrane to ensure that the membrane was positive and negative. The transfer solution should be pre-cooled, so the membrane was transferred in an ice water bath, 100 V, for 1 h. The membrane was blocked 1 h at room temperature with 5% skimmed milk blocking solution configured with TBST, incubated overnight at 4°C with the primary antibody configured with 1:1,000 blocking solution, and shaked at room temperature at low speed. The membrane was washed with TBST three times, 5 min each time. The secondary antibody was incubated for 1 h at room temperature, shaken at room temperature at low speed. The membrane was washed three times using TBST, 5 min each time. ImageQuant LAS 500 was used to perform chemiluminescence detection on PVDF membrane. ImageJ software was used to measure the gray value of PVDF membrane. A single factor was analyzed to calculate the *p* value to determine whether the group had a significant difference.

### ELISA Analysis

Fifty µL of blood was collected from each mouse, and serum was isolated from the whole blood via centrifugation at 1000 *g* for 10 min. The levels of serum SP-A, SP-B, TGF-β, and TLR4 were measured by using the ELISA kit from Sangon Biotech (Shanghai, China).

### Measurement of Oxidative Stress Parameters

The activity of superoxide dismutase (SOD) in serum was measured by using SOD assay kit. The serum level of malondialdehyde (MDA) was measured by using MDA assay kit. All kits were purchased from Sangon Biotech (Shanghai, China).

### Statistical Analysis

All data were presented as mean values ± standard deviations (S.D.). Two independent samples *t* test was used to compare the sample data between groups in this study, and *p* < 0.05 indicated that the difference was statistically significant.

## Results

### Pathological Changes of Lung Tissues in Mice After Paraquat Poisoning

**FIGURE 1:**
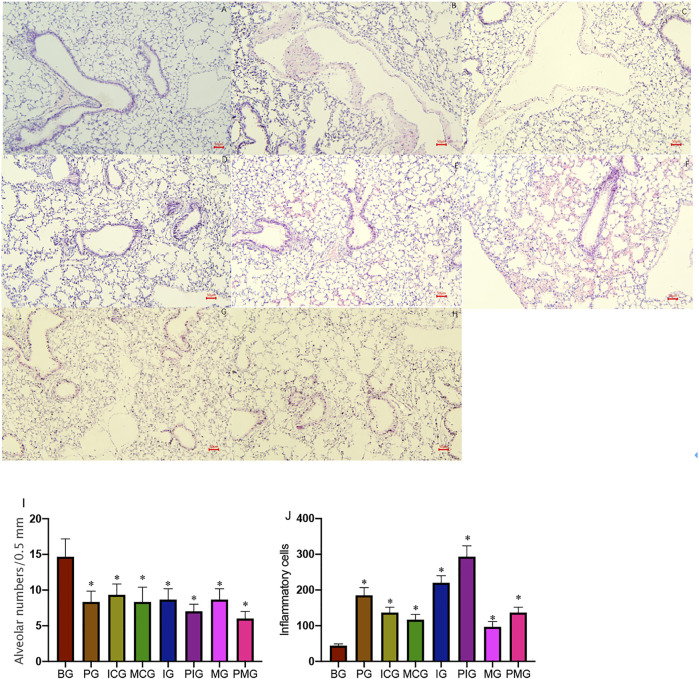
HE staining shows pathological changes of lung tissues in mice after PQ poisoning. **(A)**, blank control group (BG). **(B)**, PQ-treated group (PG, a paraquat poisoning model was established via intraperitoneal injection of paraquat). **(C)**, miR-199 siRNA control group (ICG). **(D)**, miR-199 mimic control group (MCG). **(E)**, miR-199 siRNA group (IG). **(F)**, PQ-treated miR-199 siRNA group (PIG). **(G)**, miR-199 mimic group (MG). **(H)**, PQ-treated miR-199 mimic group (PMG). **(I)**, mean linear intercept (MLI) was used to determine alveolar destruction and altered air space by overlaying a prearranged grid on the image, with set placed lines totaling 0.5 mm in length. **(J)**, quantification of inflammatory cells among different groups. *n* = 3 for each group. *p < 0.05 vs. the BG group.

After the mice were injected with paraquat 40 mg/kg in one time, four mice died without intervention on 2 days (1), 4 days (1), and 7 days (2), respectively. The results suggested that the acute lung injury period was the high incidence period of death within 7 days of PQ treatment. The results of lung HE staining showed that the cells were in perfect structure without inflammation in BG (Figure 1A). In the PG group, the destruction of the alveolar structure was observed in the tissue, bleeding in the alveolar cavity, and infiltration of a large number of inflammatory cells was observed (Figure 1B). The cells were in clear structure with a little inflammation in ICG and MCG groups (Figures 1C,D). In the IG group, it was observed that the destruction of alveolar structure was further aggravated, with thickening of local alveolar septum, diffuse hemorrhage, and a large number of inflammatory cell infiltration in the alveoli (Figure 1E). In the PIG group, it was observed that the destruction of alveolar structure was significantly aggravated, with thickening of local alveolar septum, diffuse hemorrhage, and a larger number of inflammatory cell infiltration in the alveoli (Figure 1F). In contrast, in the MG group, the destruction of the alveolar structure was less in the tissue, and infiltration of a few numbers of inflammatory cells was observed (Figure 1G). In the PMG group, the destruction of the alveolar structure was also less in the tissue, and infiltration of a few numbers of inflammatory cells was observed (Figure 1H). MLI analysis showed that alveolar number was reduced in the PG group (7–10/0.5 mm) when compared with that in the BG group (12–17/0.5 mm, Figure 1I, Supporting file 1, *p* < 0.05), suggesting that ALI model caused by PQ may be established by destroying the alveolar structure. Alveolar number was increased in the ICG (8–11/0.5 mm) and MCG groups (6–10/0.5 mm, Figure 1I, Supporting file 1). Alveolar number was reduced in the PIG group (6–8/0.5 mm) when compared with the IG groups (6–10/0.5mm, Figure 1I, Supporting file 1). Alveolar number was reduced in the PMG group (5–7/0.5 mm) when compared with the IG groups (7–10/0.5 mm, Figure 1I, Supporting file 1), PQ further caused the alveolar destruction and miR-199 might cause little change on the alveolar number. Comparatively, inflammatory cell number was increased in the PG group when compared with that in the BG group (Figure 1J, *p* < 0.05), suggesting that ALI model caused by PQ may be established by increasing inflammatory situation. Inflammatory cell number was reduced in the ICG and MCG groups when compared with the PG group (Figure 1J). Inflammatory cell number was further increased in the IG and PIG group and reduced in MG and PMG group (Figure 1J), suggesting PQ and miR-199 affect inflammatory situation in the ALI cellular model. The results suggest that PQ caused significant lung injury, which was further aggregated after miR-199 siRNA treatment while the injury could be repaired by miR-199 mimics.

### Paraquat Treatment Increased miR-199 Level and Reduced SET Level

**FIGURE 2:**
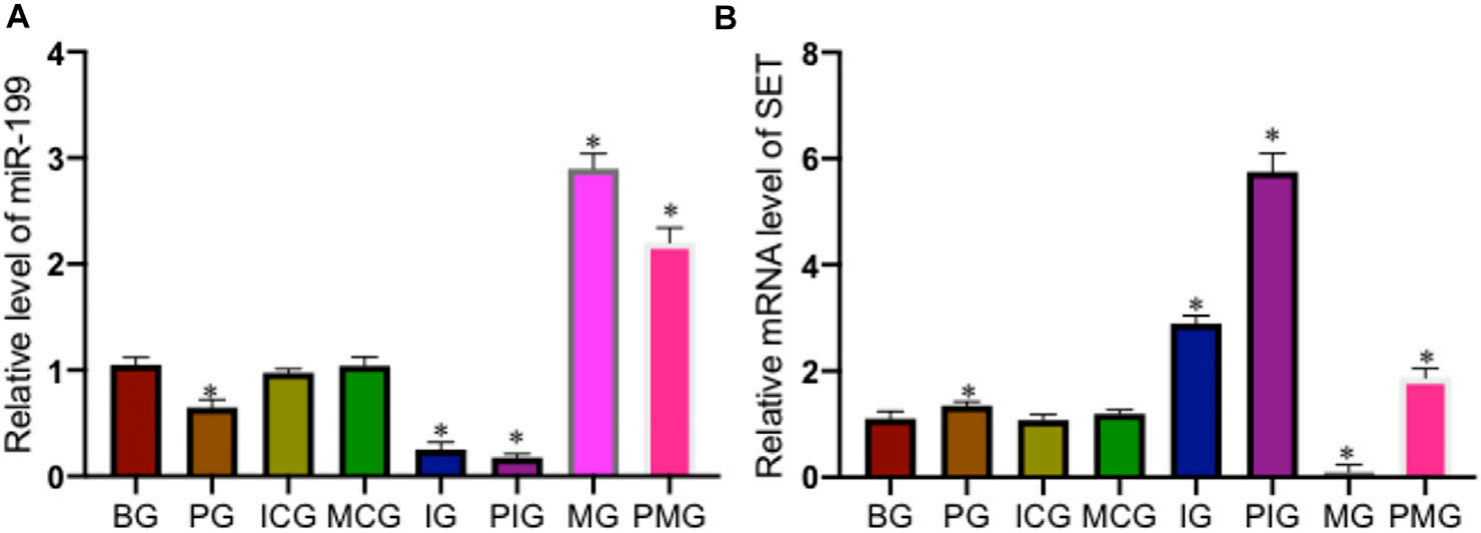
Relative level of miR-199 and relative mRNA levels of SET among different groups. **(A)**, relative level of miR-199. **(B)**, relative mRNA levels of SET. *n* = 3 for each group. *p < 0.05 vs. the BG group.

**FIGURE 3:**
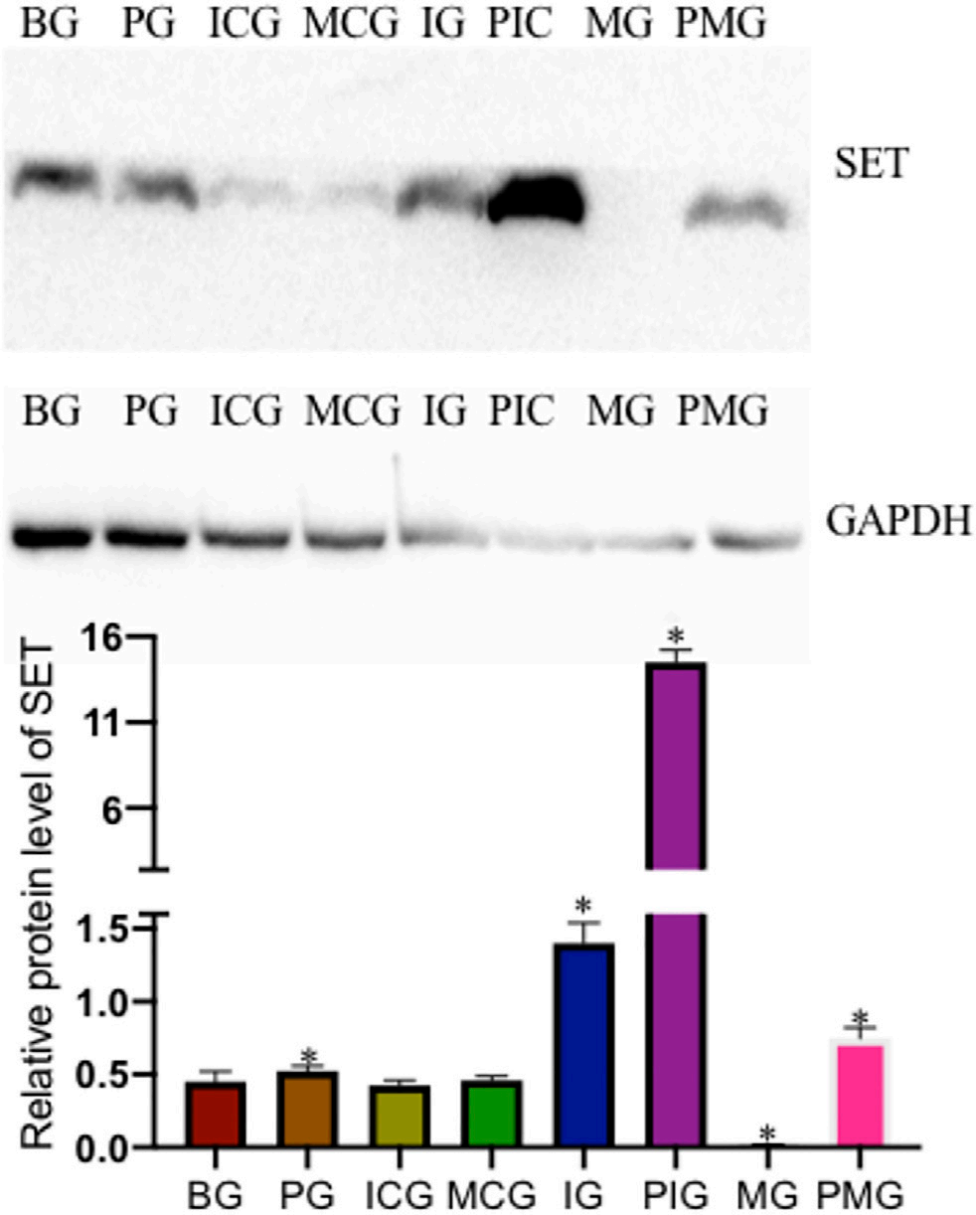
Relative protein level of SET among different groups. *n* = 3 for each group. *p < 0.05 vs. the BG group.

qPCR analysis showed that PQ treatment reduced miR-199 level in the PG group when compared with that in the BG group (Figure 2A, *p* < 0.05). miR-199 reached the lowest levels in the IG and PIG groups, and reached the highest levels in the MG and PMG groups (Figure 2A, *p* < 0.05), suggesting miR-199 was successfully silenced or overexpressed after miR-199 siRNA or mimic treatments. PQ treatment increased relative mRNA level of SET in PG group when compared with that in the BG, ICG, and MCG groups (Figure 2B, *p* < 0.05). SET mRNA reached the highest levels in the IG and PIG groups, and reached the lowest levels in the MG and PMG groups (Figure 2B, *p* < 0.05), suggesting miR-199 controlled the relative mRNA level of SET. Western Blot analysis showed the similar changing trend and PQ treatment increased relative protein level of SET in PG group when compared with that in the BG, ICG, and MCG groups (Figure 3, *p* < 0.05). SET protein reached the highest levels in the IG and PIG groups, and reached the lowest levels in the MG and PMG groups (Figure 3, *p* < 0.05), suggesting miR-199 controlled the expression of SET gene.

### Paraquat Poisoning Affected the Biomarkers of Lung Injury

**FIGURE 4:**
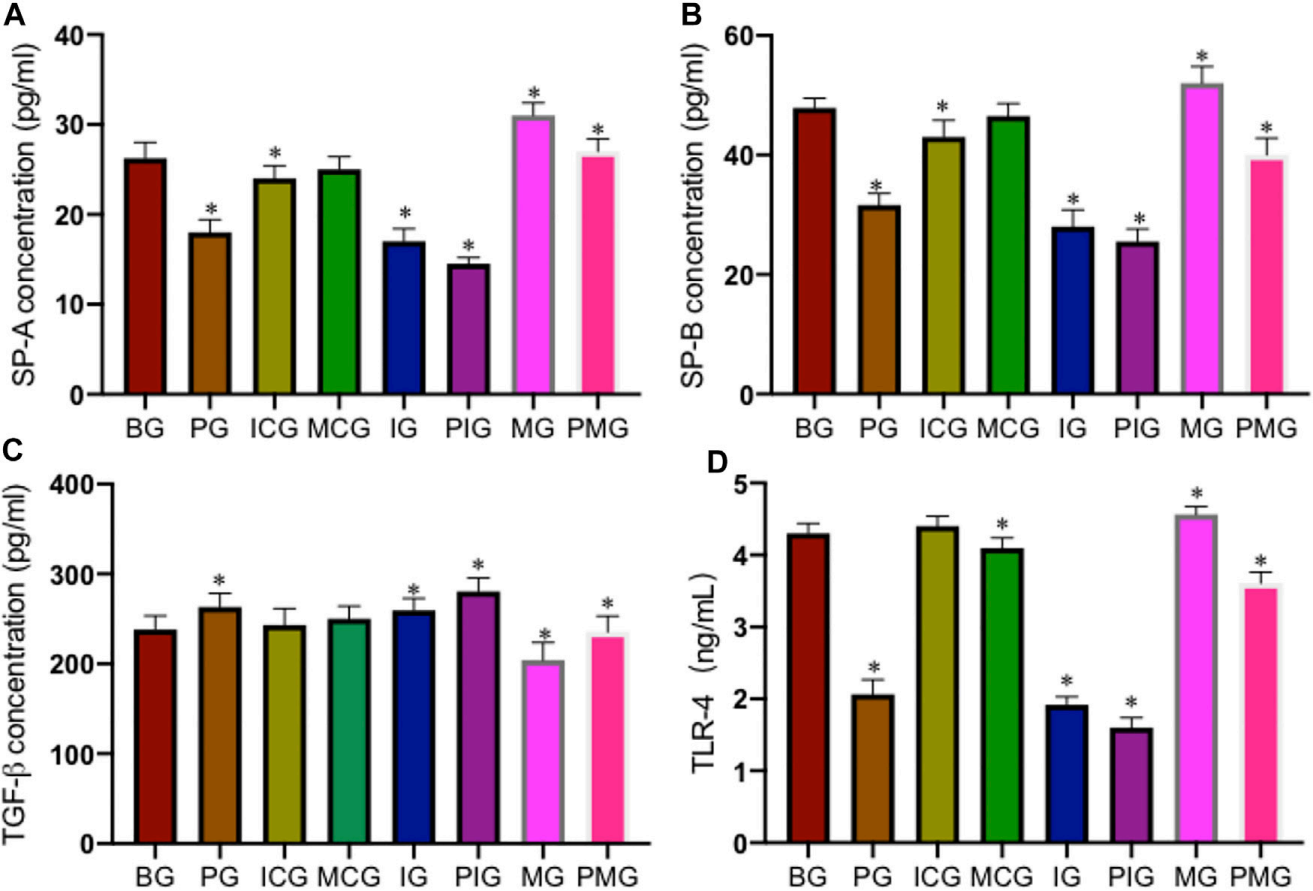
The levels of serum biomarkers of lung injury among different groups. **(A)**, SP-A concentration. **(B)**, SP-B concentration. **(C)**, TGF-β concentration. **(D)**, TLR4 concentration. *n* = 3 for each group. *p < 0.05 vs. the BG group.

ELISA analysis showed that PQ treatment reduced serum SP-A level in PG group when compared with that in the BG, ICG, and MCG groups (Figure 4A, *p* < 0.05). Serum SP-A reached the lowest levels in the IG and PIG groups, and reached the highest levels in the MG and PMG groups (Figure 4A, *p* < 0.05), suggesting miR-199 overexpression increased the level of serum SP-A. Similarly, PQ treatment reduced serum SP-B level in PG group when compared with that in the BG, ICG, and MCG groups (Figure 4B, *p* < 0.05). Serum SP-B reached the lowest levels in the IG and PIG groups, and reached the highest levels in the MG and PMG groups (Figure 4B, *p* < 0.05), also suggesting miR-199 overexpression increased the level of serum SP-B. In contrast, PQ treatment increased serum TGF-β level in the PG group when compared with that in the BG, ICG, and MCG groups (Figure 4C, *p* < 0.05). Serum TGF-β reached the highest levels in the IG and PIG groups, and reached the lowest levels in the MG and PMG groups (Figure 4C, *p* < 0.05), suggesting miR-199 overexpression reduced the level of serum TGF-β. PQ treatment reduced serum TLR4 level in the PG group when compared with that in the BG group (Figure 4D, *p* < 0.05). Serum TLR4 reached the lowest levels in the IG and PIG groups, and reached the highest levels in the MG and PMG groups (Figure 4D, *p* < 0.05), suggesting miR-199 overexpression reduced the level of serum TLR4.

### Paraquat Poisoning Increased Oxidative Stress

**FIGURE 5:**
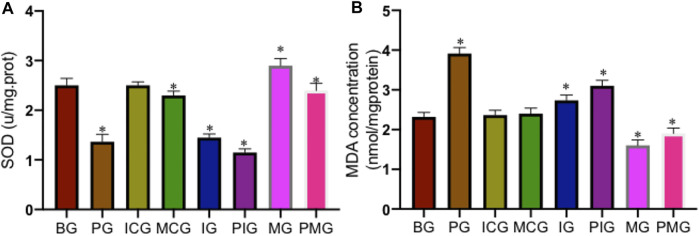
The levels of oxidative biomarkers of lung injury among different groups. **(A)**, SOD activity. **(B)**, MDA concentration. *n* = 3 for each group. *p < 0.05 vs. the BG group.

PQ treatment reduced serum SOD activity in the PG group when compared with that in the BG, ICG, and MCG groups (Figure 5A, *p* < 0.05). Serum SOD activity reached the lowest levels in the IG and PIG groups and reached the highest levels in the MG and PMG groups (Figure 5A, *p* < 0.05), also suggesting miR-199 overexpression increased the activity of serum SOD. In contrast, PQ treatment increased serum MDA concentration in PG group when compared with that in the BG, ICG, and MCG groups (Figure 5B, *p* < 0.05). Serum MDA concentration reached the highest levels in the IG and PIG groups and reached the lowest levels in the MG and PMG groups (Figure 5B, *p* < 0.05), suggesting miR-199 overexpression partially reduced the level of serum MDA concentration. All these results suggest that PQ poisoning increased oxidative stress, which could be controlled by miR-199.

## Discussion

**FIGURE 6:**
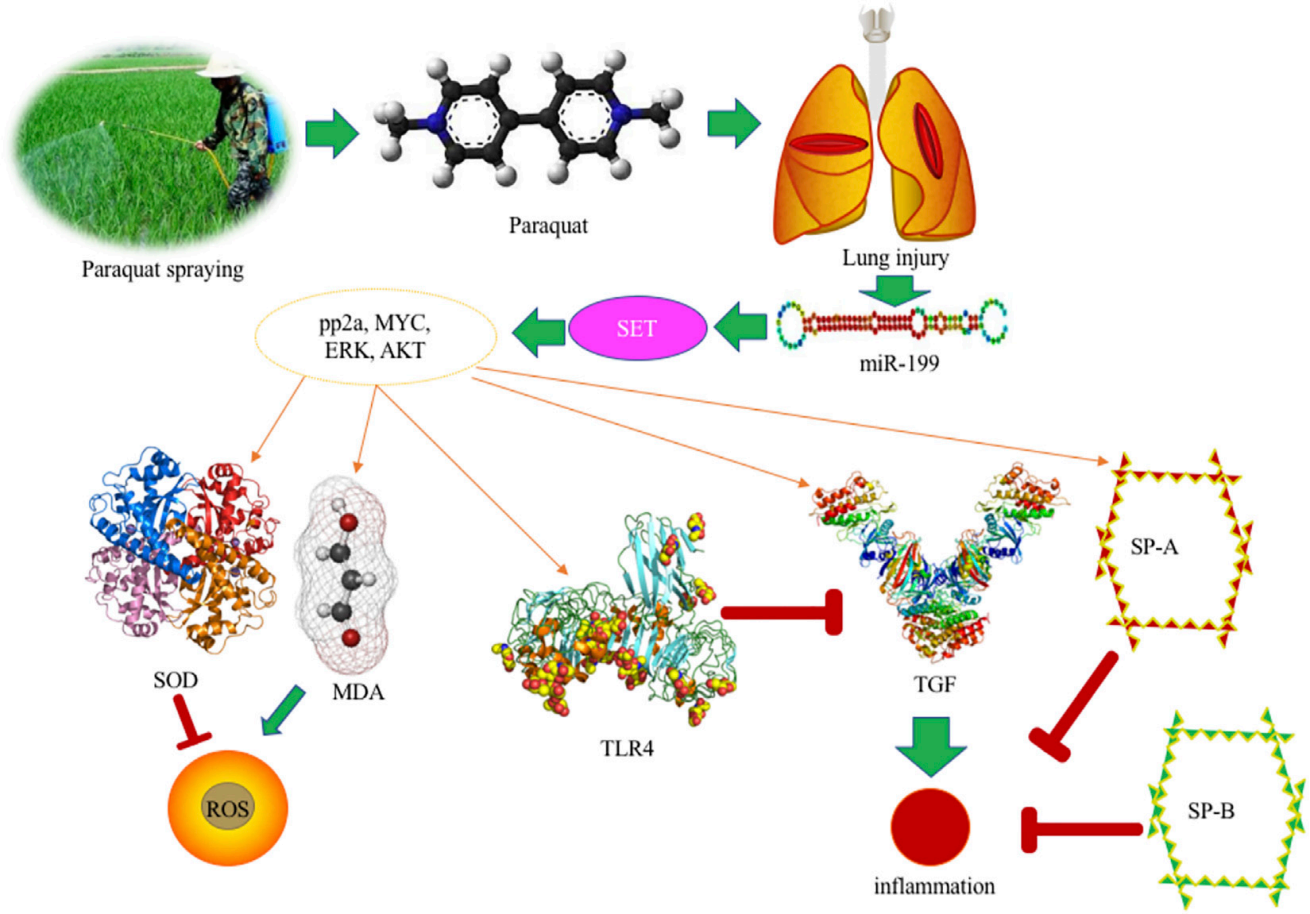
Schematic plot of possible molecular mechanisms for PQ induced lung injury.

PQ, as a toxic bipyridine compound, is currently one of the herbicides commonly used in agricultural production. However, due to its highly toxic nature, a small dose of PQ intake can have fatal consequences because of serious lung injury (Yanling et al., 2019; Subbiah and Tiwari, 2021) (Figure 6). At present, the fatality rate of PQ poison is very high, and there is lack of evidence-based recommendations and specific antidote to treat its poison. PQ poisoning becomes a serious public health problem. PQ has a highly toxic reaction to the body after ingestion, which can cause a series of complications, including acute respiratory distress, compression syndrome, pulmonary fibrosis, renal failure, liver toxicity, etc. Seeking effective poisoning drugs has become a hot and difficult point in clinical medical research. The purpose of this study is to explore the possible mechanism by using miR-199 mediated SET gene, and provide clues and directions for studying the etiology, mechanism of action, and research treatment of lung injury caused by PQ poisoning.

The study on the mechanism of PQ poisoning should use the same route of PQ exposure as the clinic. Since PQ poisoning patients are mainly oral in practice, this study uses the most commonly used route that is consistent with this, that is, one-time intraperitoneal injection administration. However, there is no uniform standard for the establishment of the PQ poisoning mice model in related studies. In this study, the two main routes of exposure are gastric gavage and intraperitoneal injection. Mice with a PQ of 40 mg/kg have the best effect on pulmonary fibrosis at 2 weeks. After a one-time intraperitoneal injection administration of paraquat, lung tissue sections were made and HE stained at 2 weeks, respectively. The results showed diffuse alveolar thickening and interstitial pulmonary fibrosis. In addition to the two conventional methods of exposure, some scholars instilled paraquat solution into the nasal cavity of C57Black/6 J mice. The results showed that the pathological changes of the lungs of the mice were very similar to those of clinical paraquat poisoning patients. And it will show typical pulmonary fibrosis at 3 weeks. Although there are differences in the dose and route of exposure in the related studies of paraquat poisoning mice, the time for typical pulmonary fibrosis to appear is concentrated in about 14 days. Therefore, in this study, the dose of 40 mg/kg was 20% PQ stock solution was diluted to 0.1 ml and then given to mice by one-time intraperitoneal injection administration, and the lung tissue samples and alveolar lavage fluid samples of mice were collected after 14 days.

Under the action of miR-199, the expression of SET protein is down-regulated, and a significant decrease in SET can be observed. Therefore, SET is considered to play an important role in PQ-mediated lung toxicity, and it is classified as a key protein for PQ-mediated lung toxicity. In this experiment, protein extraction was performed on eight groups at the same time and the subsequent Western blot showed that the expression of SET was very high in the PQ model. SET has been reported to be the inhibitor of protein phosphatase 2 A (PP2A) (Dacol et al., 2021), which is associated with kidney development and the activities of ion-transporter, aquaporin-2 and podocytes, and participates in many renal physiological processes (Shao et al., 2021). PP2A activation controls inflammation in a mouse model of acute lung injury (McHugh et al., 2016). In the present results, miR-199 overexpression controlled the inflammation in the lung injury model by reducing the expression of SET, and TGF-β concentration, and increasing the level TLR4 (Figures 3, 4). PQ can induce lung injury by modulating inflammation and oxidative stress. SP-A, SP-B, TGF-β, and TLR4 are critical meditators of inflammation in lung injury (Yang et al., 2020; Ben et al., 2012; Pittet et al., 2001), and SOD and MDA are associated with oxidative stress (Liu et al., 2019; Meng et al., 2019), and all of these molecules may be mediated by miR-199 (Figure 6), since miR-199 is involved with the regulation of inflammatory and oxidative activates (Wang et al., 2018b; Wu et al., 2020).

ERK, MYC, and AKT are the important biomarkers during lung injury by affecting inflammatory responses (Meyer et al., 2009; Schuh and Pahl, 2009; Zhang et al., 2019). Activation of the ERK pathway will induce lung injury and trigger lung inflammation (Gonçalves-de-Albuquerque et al., 2012). TLR4 plays an important role in the prevention of inflammation and lung injury (Figure 6) (Yang et al., 2012; Hu et al., 2013). Myc is also involved with programming inflammation, immune suppression, and lung injury (Figure 6) (Zhang et al., 2019; Kortlever et al., 2017). TLR4 and Akt signaling affects systemic inflammatory responses during lung injury and dysfunction (Figure 6) (Meng et al., 2018; Yan et al., 2018).

Pulmonary surfactant protein SP-A and SP-B play an important role in maintaining the normal physiological functions of the alveoli and regulating the immune balance of the local microenvironment, and are reduced after PQ treatment in the present study (Figure 4). However, the changing law of SP-A and SP-B in ALI and their regulatory factors are subjects that still need further verification. This study shows that the contents of SP-A and SPB are significantly reduced in acute lung injury. The mechanism of the decrease may be due to the fact that surfactant protein is an important component of surface substances. Alveolar type II epithelial cells secrete the surface in the form of lamellar bodies. Active substances enter the alveolar cavity, so type II epithelial cells swell, fall off, vacuole-like degeneration, lamellar emptying, or vacuole-like degeneration may be the morphological basis for the decrease in SPA and SP-B content. Secondly, refractory hypoxemia, the release of a large number of inflammatory cytokines, the release of oxygen free radicals (O2-‚NO2‚ONOO-), cytokines, etc. can affect the activity of SP-A and SP-B proteins. This will inevitably lead to a decrease in the content of SPA and SP-B. At the same time, the protease released by neutrophils may increase the degradation of SP. The exudation of a large amount of plasma protein will consume more SP-A. The overflow of a large amount of fluid in the alveolar cavity will cause SP-A and SP-B. The concentration in the alveolar cavity decreases, thereby reducing its relative content.

## Limitations

In this study, the mechanism of PQ poisoning induced lung injury in mice was studied from the perspective of miRNA-mediated gene. Based on miR-199-mediated SET, the lung tissue was also detected. Although certain results have been achieved, there are still shortcomings. In the future, the following work can be carried out in one step. However, due to the current miRNA study still being improved, there are a small part of miRNA, and many other miRNAs in the data analysis of this experiment, which are temporarily not qualitative, and further qualitative analysis is needed after the database is perfected in the future. In addition, future research can perform targeted analysis of the potential biomarkers detected this time, and can also combine other omics (such as proteomics) to verify the metabolic pathways from different levels, in order to be more in-depth and more comprehensive to clarify the mechanism of PQ poisoning-induced lung injury in mice.

The above studies show that SET has important biological effects. The current research on SET gene expression and its functional regulation mechanism is not complete enough, including the appearance of SET-related miRNA regulation, etc. The exploration of genetic regulation mechanisms, especially in lung injury, is even rarer. Therefore, the study of the SET gene in lung cancer cells may lay the foundation as the potential target for the prevention and clinical treatment of lung injury in the future.

## Conclusion

This study shows that PQ poisoning can cause lung damage in mice, mainly inflammatory response and oxidative stress. There are significant differences in the metabolic profile of lung tissue and alveolar lavage fluid in paraquat-poisoned mice. This study provided a very important theoretical basis for subsequent clinical treatment and scientific research of lung injury caused by PQ.

Supporting file 1: HE staining shows pathological changes of lung tissues in mice after PQ poisoning. A, blank control group (BG). B, PQ-treated group (PG, a paraquat poisoning model was established via intraperitoneal injection of paraquat). C, miR-199 siRNA control group (ICG). D, miR-199 mimic control group (MCG). E, miR-199 siRNA group (IG). F, PQ-treated miR-199 siRNA group (PIG). G, miR-199 mimic group (MG). H, PQ-treated miR-199 mimic group (PMG). Mean linear intercept (MLI) was used to determine alveolar destruction and altered air space by overlaying a prearranged grid on the image, with set placed lines totaling 0.5 mm in length.

## Data Availability

The original contributions presented in the study are included in the article/Supplementary Material, further inquiries can be directed to the corresponding author.

## References

[B1] Ben D. F. Yu X. Y. Ji G. Y. Zheng D. Y. Lv K. Y. Ma B. (2012). TLR4 Mediates Lung Injury and Inflammation in Intestinal Ischemia-Reperfusion. J. Surg. Res. 174 (2), 326–333. 10.1016/j.jss.2010.12.005 21392794

[B2] Bi Y. Zhu X. Yu Z. Yi M. Han X. Ren J. (2020). Clinical Outcomes of Self-Expandable Metallic Stents for Malignant Obstructive Atelectasis. Sci. Rep. 10 (1), 3600–3607. 10.1038/s41598-020-60566-6 32107423 PMC7046663

[B3] Chao A. Tsai C. L. Wei P. C. Hsueh S. Chao A. S. Wang C. J. (2010). Decreased Expression of microRNA-199b Increases Protein Levels of SET (Protein Phosphatase 2A Inhibitor) in Human Choriocarcinoma. Cancer Lett. 291 (1), 99–107. 10.1016/j.canlet.2009.10.005 19900756

[B4] Chernikov I. V. Gladkikh D. V. Meschaninova M. I. Ven'yaminova A. G. Zenkova M. A. Vlassov V. V. (2017). Cholesterol-containing Nuclease-Resistant siRNA Accumulates in Tumors in a Carrier-free Mode and Silences MDR1 Gene. Mol. Ther. Nucleic Acids 6, 209–220. 10.1016/j.omtn.2016.12.011 28325287 PMC5363506

[B5] Corrêa M. P. Andrade F. E. C. Gimenes A. D. Gil C. D. (2017). Anti-inflammatory Effect of Galectin-1 in a Murine Model of Atopic Dermatitis. J. Mol. Med. (Berl) 95 (9), 1005–1015. 10.1007/s00109-017-1566-9 28664215

[B6] Dacol E. C. Wang S. Chen Y. Lepique A. P. (2021). The Interaction of SET and Protein Phosphatase 2A as Target for Cancer Therapy. Biochim. Biophys. Acta (Bba) - Rev. Cancer 1876, 188578. 10.1016/j.bbcan.2021.188578 34116173

[B7] Feng Y. Li X. Zhou W. Lou D. Huang D. Li Y. (2017). Regulation of SET Gene Expression by NFkB. Mol. Neurobiol. 54 (6), 4477–4485. 10.1007/s12035-016-9967-2 27351675

[B8] Gonçalves-de-Albuquerque C. F. Silva A. R. Burth P. de Moraes I. M. M. Oliveira F. Md. J. Younes-Ibrahim M. (2012). Oleic Acid Induces Lung Injury in Mice through Activation of the ERK Pathway. London, United Kingdom: Mediators of Inflammation. 10.1155/2012/956509 PMC3504460 23209347

[B9] Hu R. Xu H. Jiang H. Zhang Y. Sun Y. (2013). The Role of TLR4 in the Pathogenesis of Indirect Acute Lung Injury. Front. Biosci. (Landmark Ed. 18 (1), 1244–1255. 10.2741/4176 23747880

[B10] Huang M. Lou D. Cai Q. Chang X. Wang X. Zhou Z. (2014). Characterization of Paraquat-Induced miRNA Profiling Response in hNPCs Undergoing Proliferation. Int. J. Mol. Sci. 15 (10), 18422–18436. 10.3390/ijms151018422 25314302 PMC4227223

[B11] Jiang C. Zhong R. Zhang J. Wang X. Ding G. Xiao W. (2019). Reduning Injection Ameliorates Paraquat-Induced Acute Lung Injury by Regulating AMPK/MAPK/NF-κB Signaling. J. Cell Biochem 120 (8), 12713–12723. 10.1002/jcb.28540 30861187

[B12] Jiang Z. F. Zhang L. Shen J. (2020). MicroRNA: Potential Biomarker and Target of Therapy in Acute Lung Injury. Hum. Exp. Toxicol. 39 (11), 1429–1442. 10.1177/0960327120926254 32495695

[B13] Johnson D. C. Chirumamilla S. K. Paez A. P. (2020). Respiratory Candida in Patients with Bronchitis, Mucus Plugging, and Atelectasis. Open Respir. Med. J. 14, 87–92. 10.2174/1874306402014010087 33717368 PMC7931156

[B14] Kortlever R. M. Sodir N. M. Wilson C. H. Burkhart D. L. Pellegrinet L. Brown Swigart L. (2017). Myc Cooperates with Ras by Programming Inflammation and Immune Suppression. Cell 171 (6), 1301–e14. e14. 10.1016/j.cell.2017.11.013 29195074 PMC5720393

[B15] Liang X. Bao X. Chen G. (2021). SET Protein in Cancer: A Potential Therapeutic Target. Mini Rev. Med. Chem. 21 (16), 2290–2299. 10.2174/1389557521666210114163318 33459234

[B16] Lin X. H. Pan H. Y. Cheng F. J. Huang K. C. Li C. J. Chen C. C. (2021). Association between liberal Oxygen Therapy and Mortality in Patients with Paraquat Poisoning: A Multi-center Retrospective Cohort Study. Plos one 16 (1), e0245363. 10.1371/journal.pone.0245363 33449962 PMC7810293

[B17] Liu B. Chen A. Lan J. Ren L. Wei Y. Gao L. (2019). Protective Mechanism of 1-methylhydantoin against Lung Injury Induced by Paraquat Poisoning. PLoS One 14 (9), e0222521. 10.1371/journal.pone.0222521 31560695 PMC6764654

[B18] Liu Y. Guan H. Zhang J. L. Zheng Z. Wang H. T. Tao K. (2018). Acute Downregulation of miR-199a Attenuates Sepsis-Induced Acute Lung Injury by Targeting SIRT1. Am. J. Physiol. Cell Physiol 314 (4), C449–C55. 10.1152/ajpcell.00173.2017 29351405

[B19] Liu Z. Qu M. Yu L. Song P. Chang Y. (2018). Artesunate Inhibits Renal Ischemia-Reperfusion-Mediated Remote Lung Inflammation through Attenuating ROS-Induced Activation of NLRP3 Inflammasome. Inflammation 41 (4), 1546–1556. 10.1007/s10753-018-0801-z 29730819

[B20] McHugh W. M. Russell W. W. Fleszar A. J. Rodenhouse P. E. Rietberg S. P. Sun L. (2016). Protein Phosphatase 2A Activation Attenuates Inflammation in Murine Models of Acute Lung Injury. Am. J. Physiol. Lung Cell Mol Physiol 311 (5), L903–L12. 10.1152/ajplung.00007.2016 27638902 PMC5130532

[B21] Meng L. Li L. Lu S. Li K. Su Z. Wang Y. (2018). The Protective Effect of Dexmedetomidine on LPS-Induced Acute Lung Injury through the HMGB1-Mediated TLR4/NF-Κb and PI3K/Akt/mTOR Pathways. Mol. Immunol. 94, 7–17. 10.1016/j.molimm.2017.12.008 29241031

[B22] Meng X. Hu L. Li W. (2019). Baicalin Ameliorates Lipopolysaccharide-Induced Acute Lung Injury in Mice by Suppressing Oxidative Stress and Inflammation via the Activation of the Nrf2-Mediated HO-1 Signaling Pathway. Naunyn Schmiedebergs Arch. Pharmacol. 392 (11), 1421–1433. 10.1007/s00210-019-01680-9 31273392

[B23] Meyer N. J. Huang Y. Singleton P. A. Sammani S. Moitra J. Evenoski C. L. (2009). GADD45a Is a Novel Candidate Gene in Inflammatory Lung Injury via Influences on Akt Signaling. FASEB J. 23 (5), 1325–1337. 10.1096/fj.08-119073 19124556 PMC2669422

[B24] Mirzaee S. Mansouri E. Shirani M. Zeinvand-Lorestani M. Khodayar M. J. (2019). Diosmin Ameliorative Effects on Oxidative Stress and Fibrosis in Paraquat-Induced Lung Injury in Mice. Environ. Sci. Pollut. Res. Int. 26 (36), 36468–36477. 10.1007/s11356-019-06572-2 31732951

[B25] Park J. Jeong S. Park K. Yang K. Shin S. (2018). Expression Profile of microRNAs Following Bone Marrow-Derived Mesenchymal Stem Cell Treatment in Lipopolysaccharide-Induced Acute Lung Injury. Exp. Ther. Med. 15 (6), 5495–5502. 10.3892/etm.2018.6118 29904430 PMC5996665

[B26] Pittet J. F. Griffiths M. J. Geiser T. Kaminski N. Dalton S. L. Huang X. (2001). TGF-beta Is a Critical Mediator of Acute Lung Injury. J. Clin. Invest. 107 (12), 1537–1544. 10.1172/JCI11963 11413161 PMC200192

[B27] Pourgholamhossein F. Rasooli R. Pournamdari M. Pourgholi L. Samareh-Fekri M. Ghazi-Khansari M. (2018). Pirfenidone Protects against Paraquat-Induced Lung Injury and Fibrosis in Mice by Modulation of Inflammation, Oxidative Stress, and Gene Expression. Food Chem. Toxicol. 112, 39–46. 10.1016/j.fct.2017.12.034 29273418

[B28] Pua Z. J. Stonestreet B. S. Cullen A. Shahsafaei A. Sadowska G. B. Sunday M. E. (2005). Histochemical Analyses of Altered Fetal Lung Development Following Single vs Multiple Courses of Antenatal Steroids. J. Histochem. Cytochem. 53 (12), 1469–1479. 10.1369/jhc.5A6721.2005 15956023 PMC3957547

[B29] Rashid A. Zeng C. Motta Ribeiro G. Hinoshita T. Lessa M. Libermann T. (2020). “Proteomics of Atelectatic versus Normally Aerated Lung in Early Endotoxemic Lung Injury,” in B67 Understanding Lung Injury: From Gene to Dysfunction (American Thoracic Society), A4101. 10.1164/ajrccm-conference.2020.201.1_meetingabstracts.a4101

[B30] Schuh K. Pahl A. (2009). Inhibition of the MAP Kinase ERK Protects from Lipopolysaccharide-Induced Lung Injury. Biochem. Pharmacol. 77 (12), 1827–1834. 10.1016/j.bcp.2009.03.012 19428337

[B31] Shao L. Ma Y. Fang Q. Huang Z. Wan S. Wang J. (2021). Role of Protein Phosphatase 2A in Kidney Disease (Review). Exp. Ther. Med. 22 (5), 1236. Epub 20210831; PubMed PMID: 34539832; PubMed Central PMCID: PMCPMC8438693. 10.3892/etm.2021.10671 34539832 PMC8438693

[B32] Sittipunt C. (2005). Paraquat Poisoning. Respir. Care 50 (3), 383–385. 15779152

[B33] SreeHarsha N. (2020). Embelin Impact on Paraquat-Induced Lung Injury through Suppressing Oxidative Stress, Inflammatory cascade, and MAPK/NF-κB Signaling Pathway. J. Biochem. Mol. Toxicol. 34 (4), e22456. 10.1002/jbt.22456 32020686

[B34] Subbiah R. Tiwari R. R. (2021). The Herbicide Paraquat-Induced Molecular Mechanisms in the Development of Acute Lung Injury and Lung Fibrosis. Crit. Rev. Toxicol. 51 (1), 36–64. 10.1080/10408444.2020.1864721 33528289

[B35] Tianzhu Z. Shihai Y. Juan D. (2014). The Effects of Morin on Lipopolysaccharide-Induced Acute Lung Injury by Suppressing the Lung NLRP3 Inflammasome. Inflammation 37 (6), 1976–1983. 10.1007/s10753-014-9930-1 24849134

[B36] Tsen C. M. Yu C. W. Chuang W. C. Chen M. J. Lin S. K. Shyu T. H. (2019). A Simple Approach for the Ultrasensitive Detection of Paraquat Residue in Adzuki Beans by Surface-Enhanced Raman Scattering. Analyst 144 (2), 426–438. 10.1039/c8an01845f 30569916

[B37] Wang Q. Zhan Y. Ren N. Wang Z. Zhang Q. Wu S. (2018). Paraquat and MPTP Alter microRNA Expression Profiles, and Downregulated Expression of miR-17-5p Contributes to PQ-Induced Dopaminergic Neurodegeneration. J. Appl. Toxicol. 38 (5), 665–677. 10.1002/jat.3571 29250806

[B38] Wang W. Guo Z. Yang S. Wang H. Ding W. (2018). Upregulation of miR-199 Attenuates TNF-α-Induced Human Nucleus Pulposus Cell Apoptosis by Downregulating MAP3K5. Biochem. Biophys. Res. Commun. 505 (3), 917–924. 10.1016/j.bbrc.2018.09.194 30309653

[B39] Wang Y. Ding L. Li Z. Chen G. Sun M. Oupicky D. (2019). Treatment of Acute Lung Injury and Early- and Late-Stage Pulmonary Fibrosis with Combination Emulsion siRNA Polyplexes. J. Control. Release 314, 12–24. 10.1016/j.jconrel.2019.10.030 31644934

[B40] Weng C. H. Chen H. H. Hu C. C. Huang W. H. Hsu C. W. Fu J. F. (2017). Predictors of Acute Kidney Injury after Paraquat Intoxication. Oncotarget 8 (31), 51345–51354. 10.18632/oncotarget.17975 28881652 PMC5584253

[B41] Wu L. Xi Y. Kong Q. (2020). Dexmedetomidine Protects PC12 Cells from Oxidative Damage through Regulation of miR-199a/HIF-1α. Artif. Cell Nanomed Biotechnol 48 (1), 506–514. 10.1080/21691401.2020.1716780 32024386

[B42] Yan J. Li J. Zhang L. Sun Y. Jiang J. Huang Y. (2018). Nrf2 Protects against Acute Lung Injury and Inflammation by Modulating TLR4 and Akt Signaling. Free Radic. Biol. Med. 121, 78–85. 10.1016/j.freeradbiomed.2018.04.557 29678610

[B43] Yang H. Z. Wang J. P. Mi S. Liu H. Z. Cui B. Yan H. M. (2012). TLR4 Activity Is Required in the Resolution of Pulmonary Inflammation and Fibrosis after Acute and Chronic Lung Injury. Am. J. Pathol. 180 (1), 275–292. 10.1016/j.ajpath.2011.09.019 22062220

[B44] Yang Y. Li Q. Tan F. Zhang J. Zhu W. (2020). Mechanism of IL-8-induced Acute Lung Injury through Pulmonary Surfactant Proteins A and B. Exp. Ther. Med. 19 (1), 287–293. 10.3892/etm.2019.8192 31853301 PMC6909794

[B45] Yanling W. Duo G. Zuojun G. Zhongqiang S. Yankai W. Shan L. (2019). Radiomics Nomogram Analyses for Differentiating Pneumonia and Acute Paraquat Lung Injury. Sci. Rep. 9 (1), 15029–9. 10.1038/s41598-019-50886-7 31636276 PMC6803642

[B46] Yeh Y. T. Chen C. K. Lin C. C. Chang C. M. Lan K. P. How C. K. (2020). Does Hemoperfusion Increase Survival in Acute Paraquat Poisoning? A Retrospective Multicenter Study. Toxics 8 (4), 84. 10.3390/toxics8040084 PMC7711471 33050540

[B47] Zhang Y. Huang T. Jiang L. Gao J. Yu D. Ge Y. (2019). MCP-induced Protein 1 Attenuates Sepsis-Induced Acute Lung Injury by Modulating Macrophage Polarization via the JNK/c-Myc Pathway. Int. Immunopharmacol 75, 105741. 10.1016/j.intimp.2019.105741 31323531

