# Supplementary figures and images for: Paraquat Induces Lung Injury via miR-199-Mediated SET in a Mouse Model
Source: Front Pharmacol. 2022 Apr 1;13:856441. doi: 10.3389/fphar.2022.856441 (PMC9011139; doi:10.3389/fphar.2022.856441)

## Slide 1
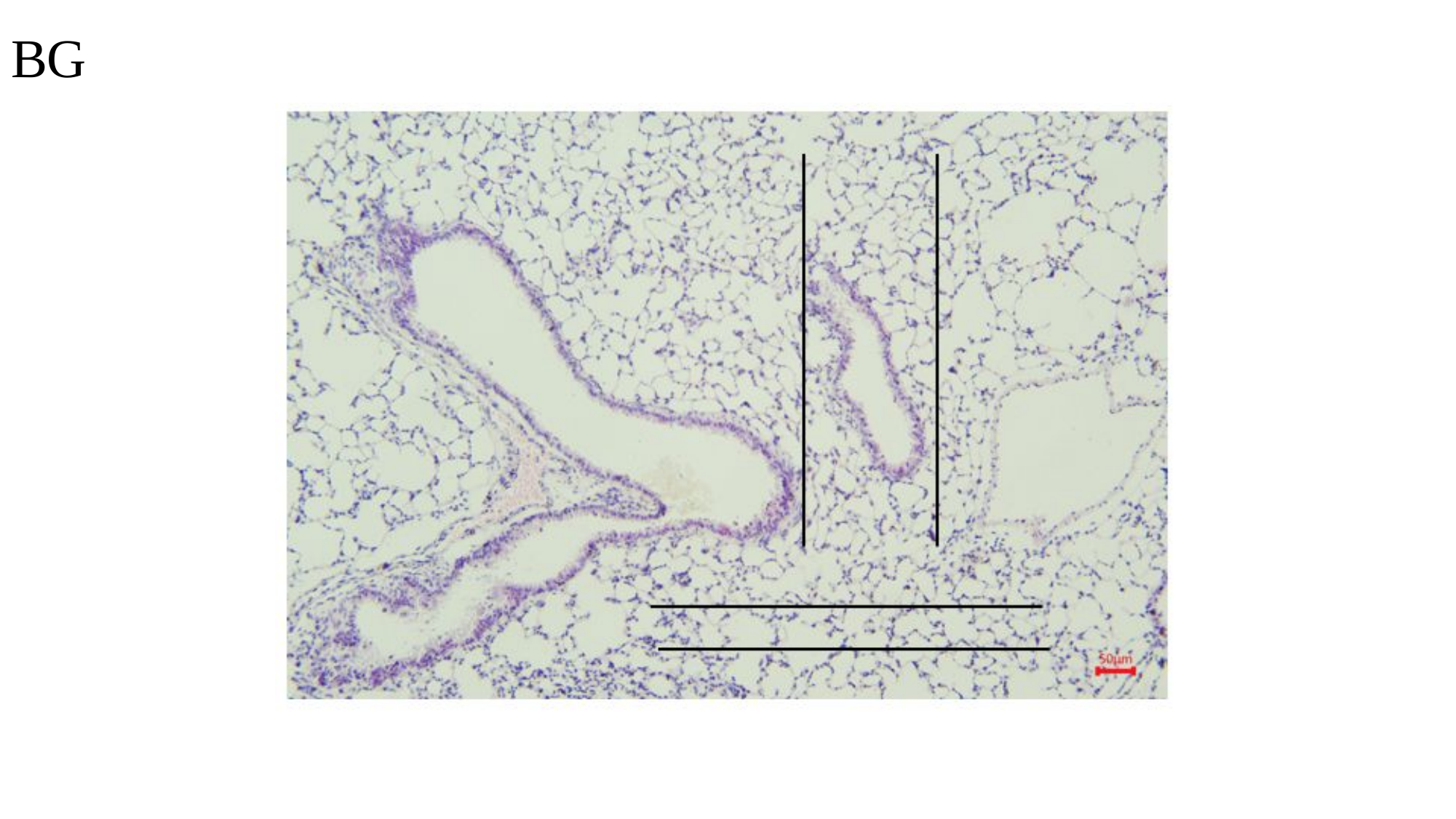

# BG

## Slide 2
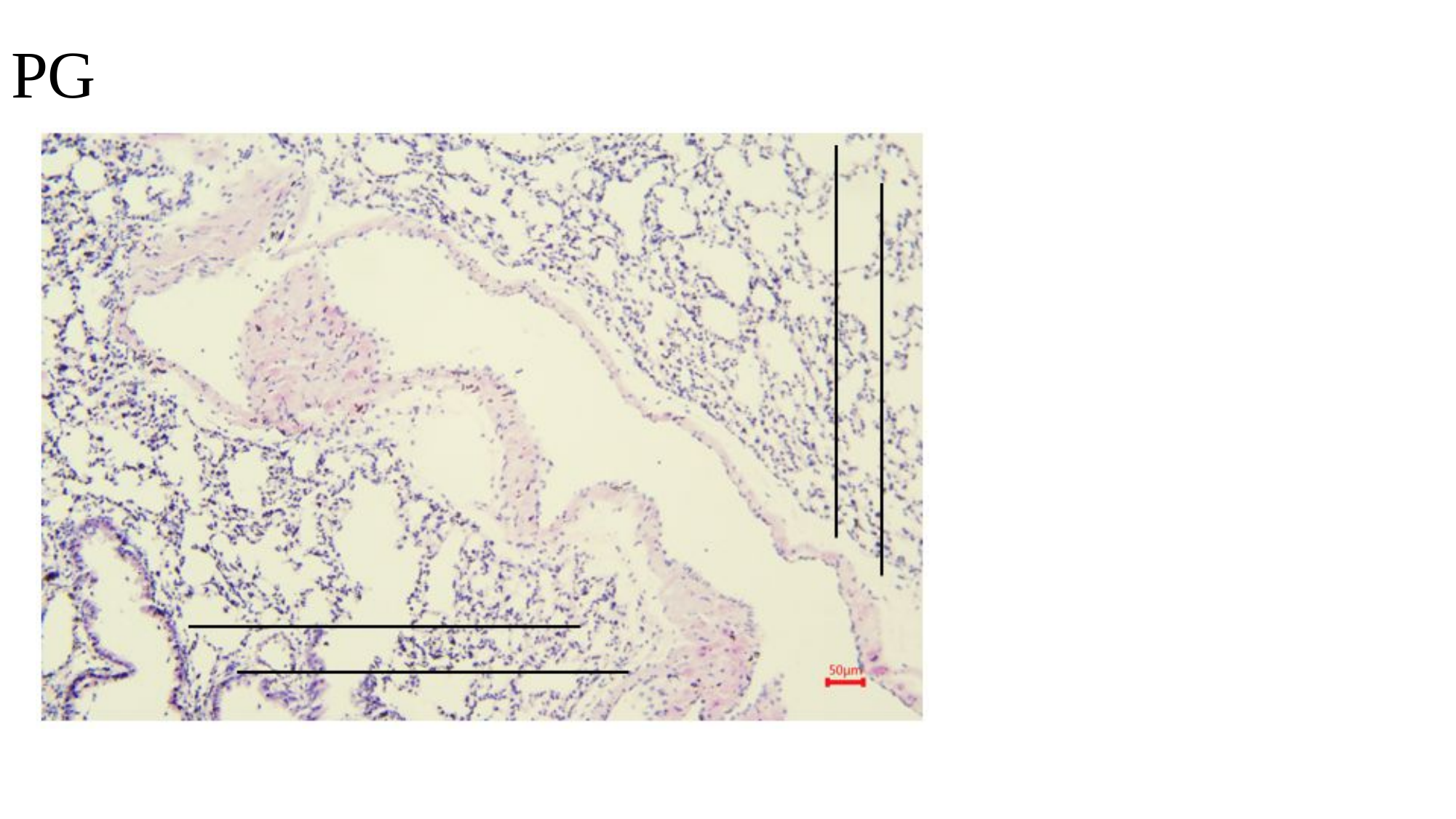

# PG

## Slide 3
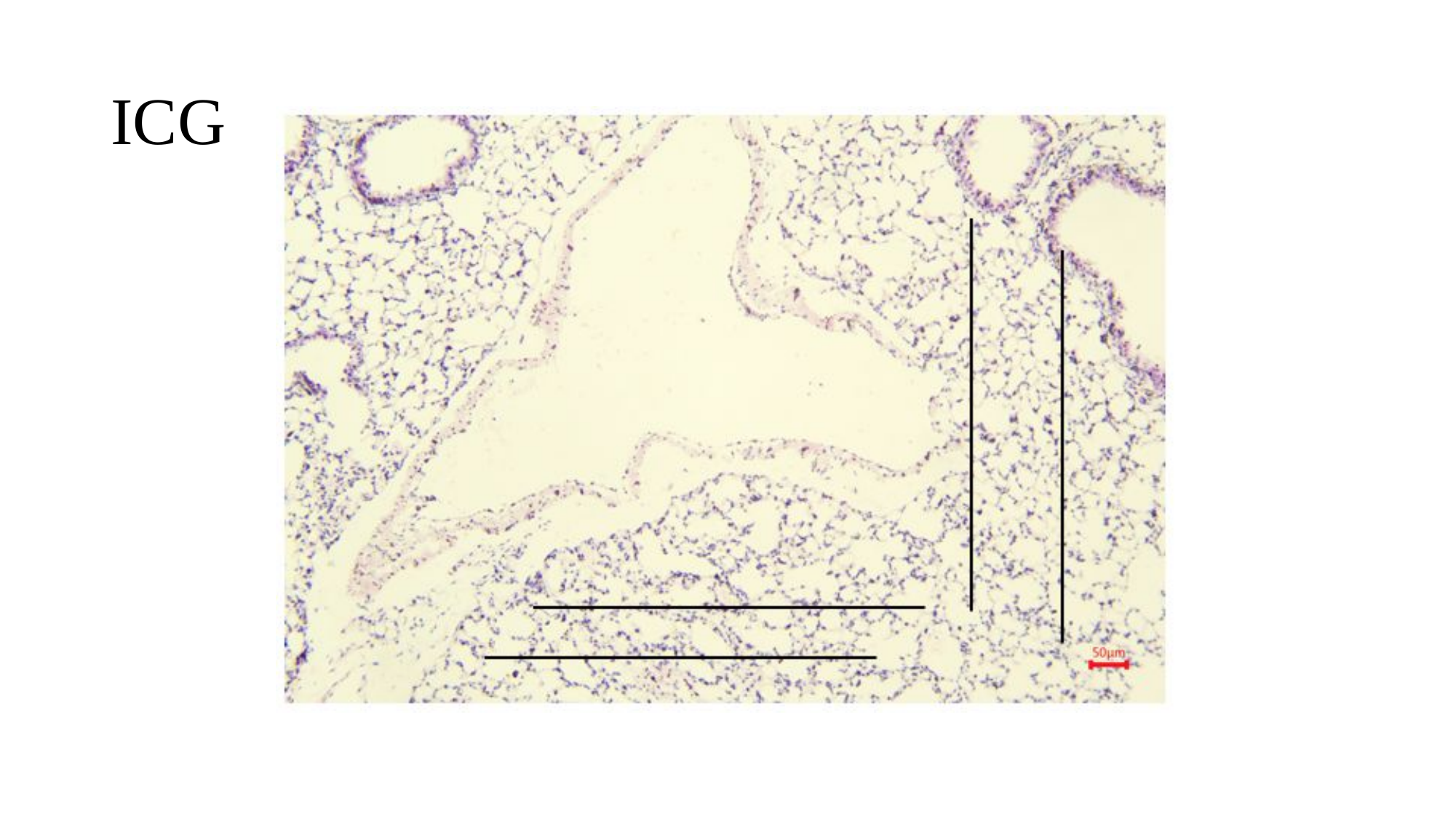

# ICG

## Slide 4
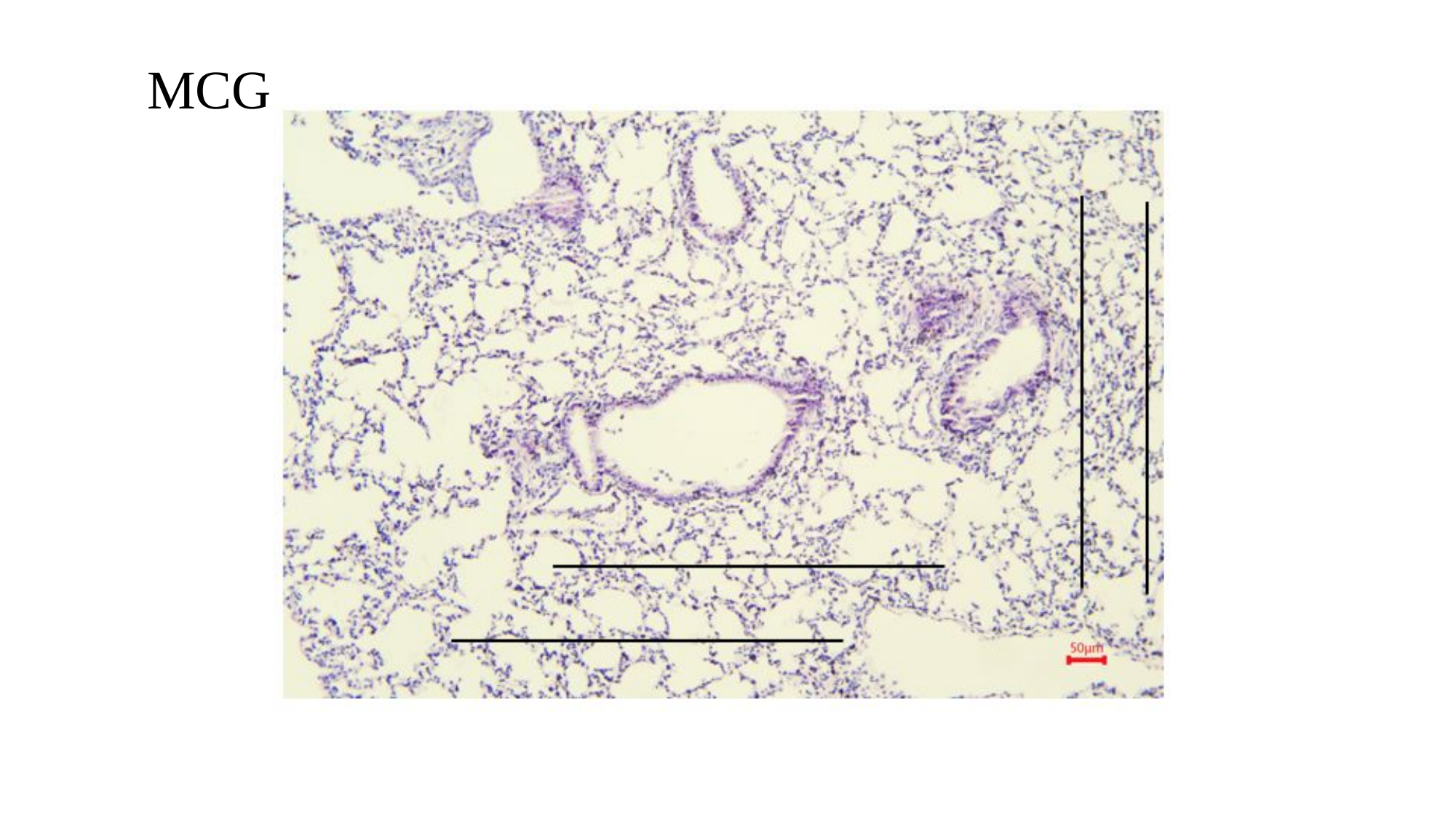

MCG

## Slide 5
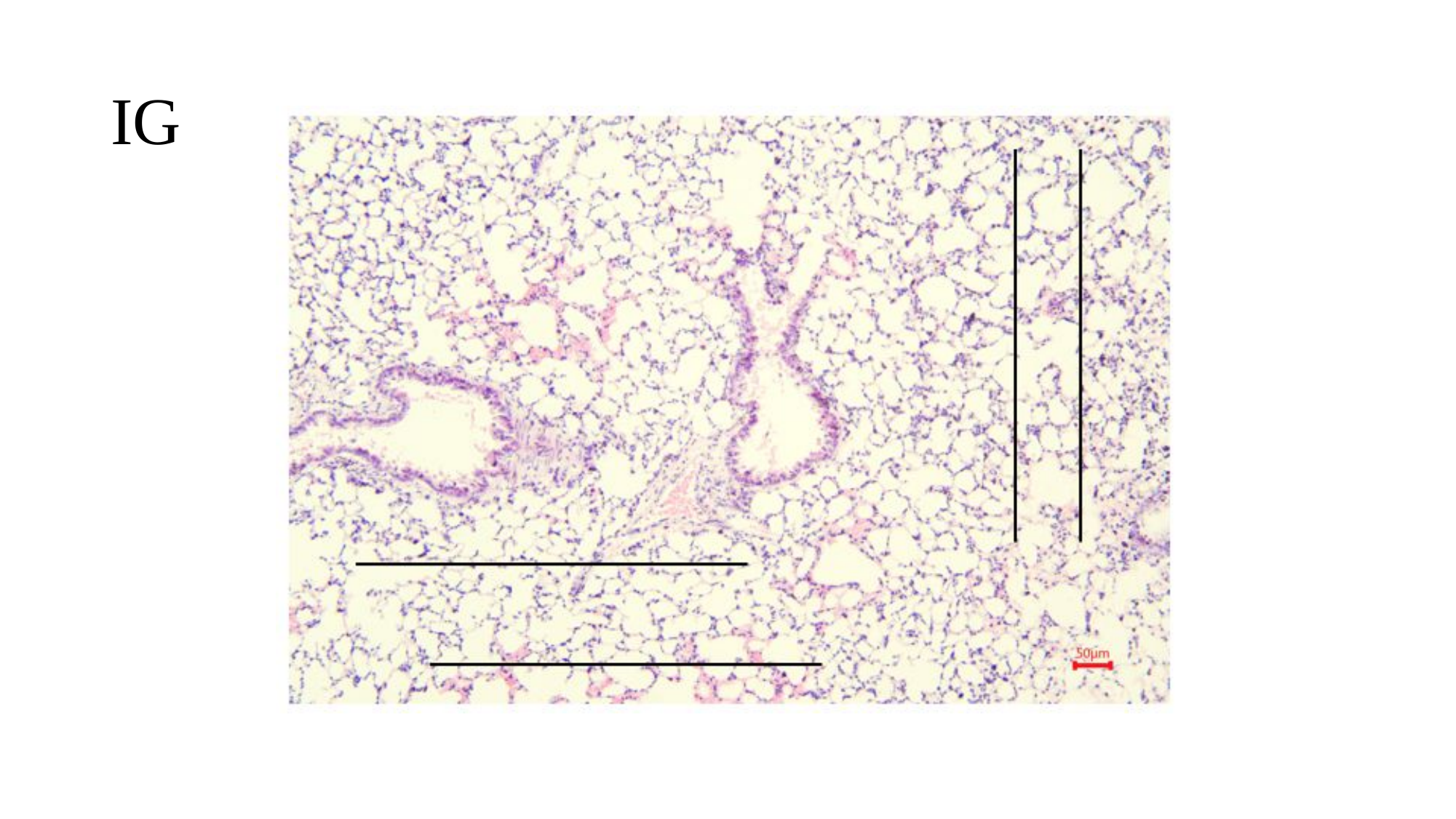

# IG

## Slide 6
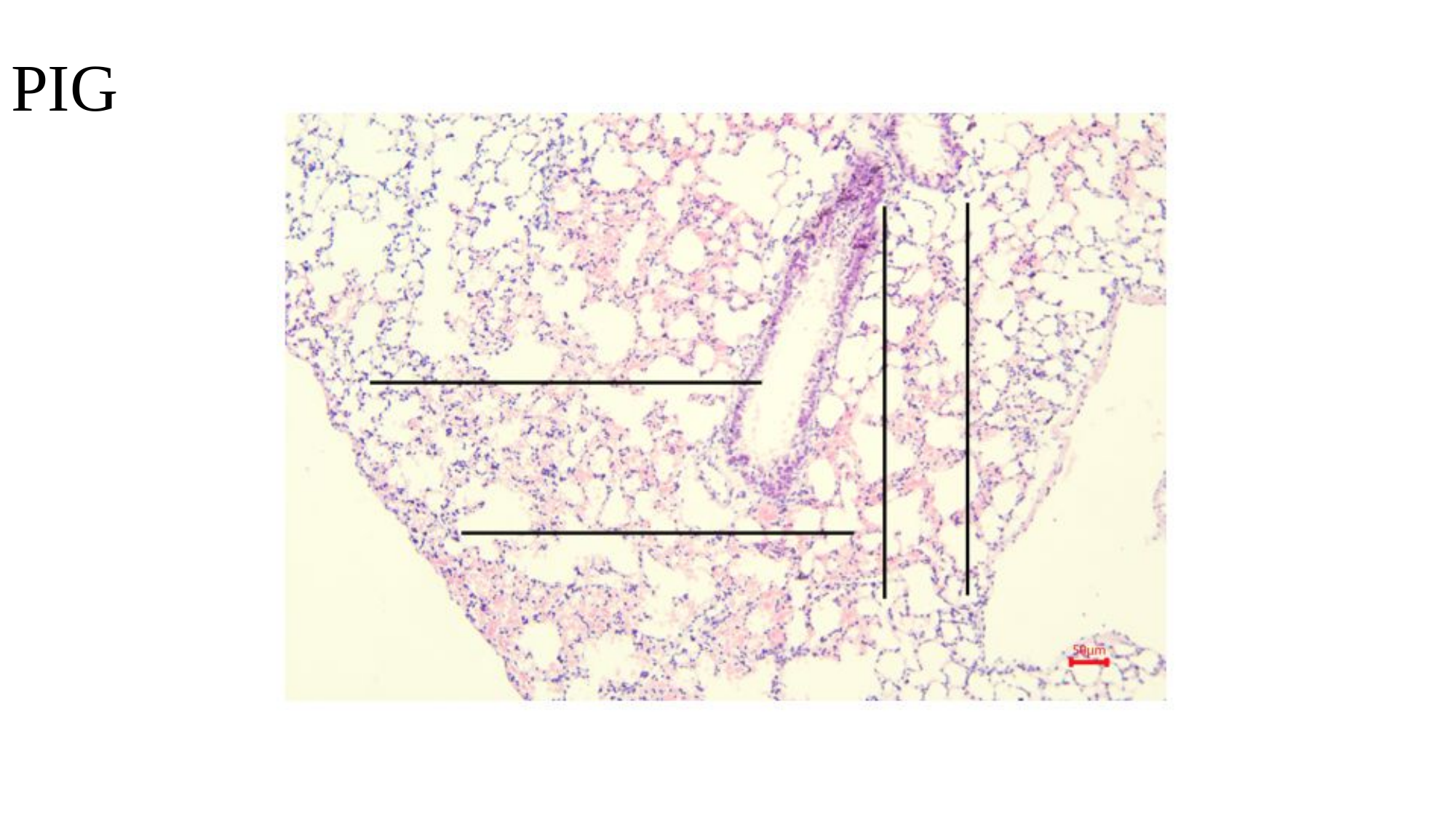

# PIG

## Slide 7
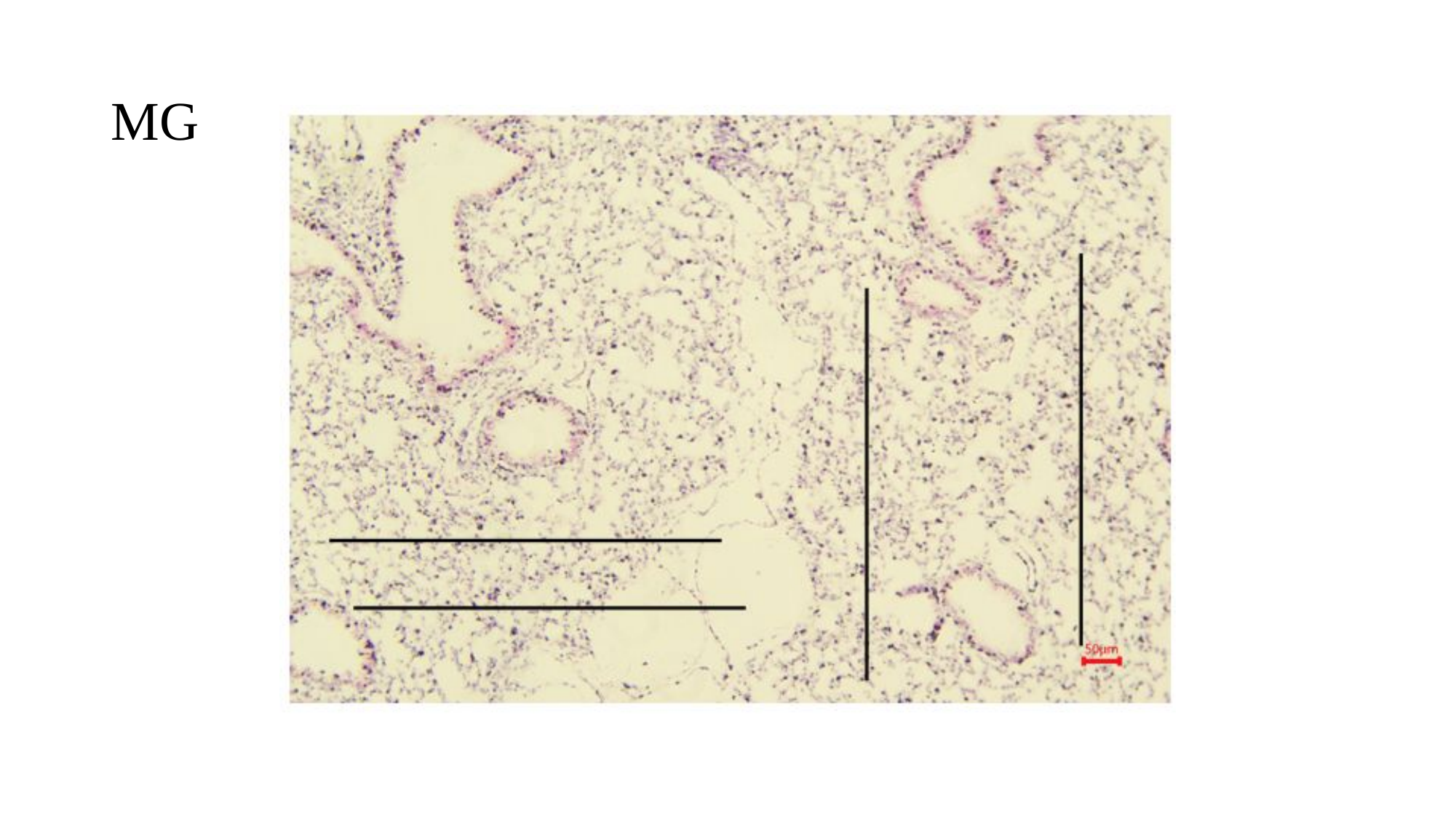

# MG

## Slide 8
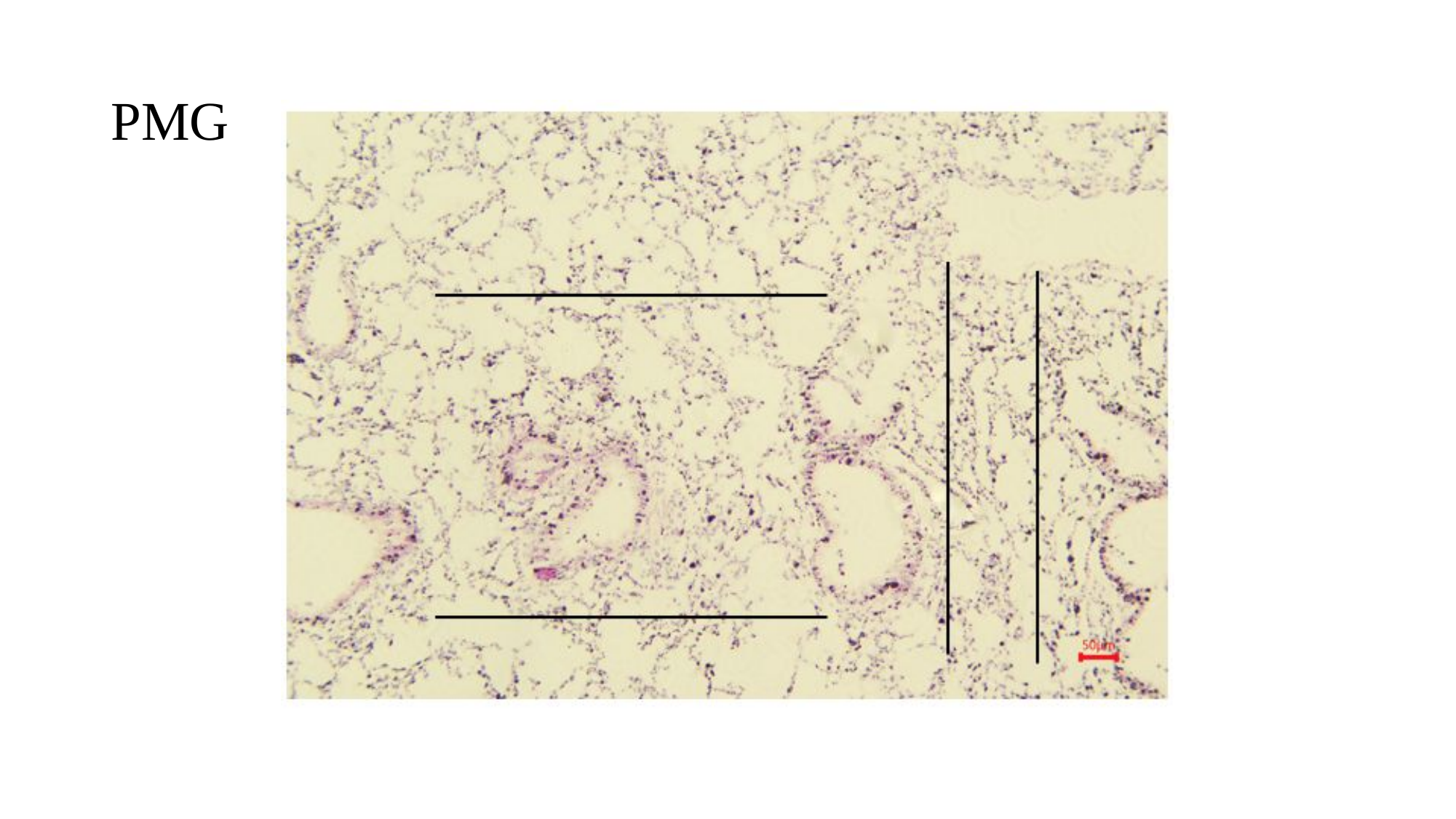

# PMG

Supplement: Supplementary file 1 [file Presentation1.pptx]
